# Leg‐type form of idiopathic multicentric Castleman disease associated with severe lower extremity chronic venous/lymphatic disease

**DOI:** 10.1002/jha2.353

**Published:** 2021-12-23

**Authors:** Thomas Ballul, Nabil Belfeki, Adèle de Masson, Véronique Meignin, Paul‐Louis Woerther, Antoine Martin, Elsa Poullot, Alain Wargnier, Jehane Fadlallah, Margaux Garzaro, Marion Malphettes, Claire Fieschi, Lucas Maisonobe, Hayat Bensekhri, Hélène Guillot, Rémi Bertinchamp, Marie Jachiet, Justine Poirot, Lionel Galicier, Eric Oksenhendler, David Boutboul

**Affiliations:** ^1^ Clinical Immunology Department Hôpital Saint Louis, Université de Paris Paris France; ^2^ Internal Medicine Department Centre hospitalier de Melun Melun France; ^3^ Dermatology Department Université de Paris Paris France; ^4^ Pathology Department Hôpital Saint Louis Université de Paris Paris France; ^5^ Bacteriology and Virology Department Hôpital Henri Mondor Créteil France; ^6^ Pathology Department Hôpital Avicenne Université Sorbonne Paris Nord Bobigny France; ^7^ Pathology Department Hôpital Henri Mondor Université Paris‐Est Créteil Créteil France; ^8^ Bacteriology Department Hôpital Saint Louis Université de Paris Paris France; ^9^ Internal Medicine Department Groupe Hospitalier Nord Essonne Longjumeau France; ^10^ Internal Medicine Department Hôpital Robert Ballanger Université Sorbonne Paris Nord Aulnay‐Sous‐Bois France; ^11^ National Reference Center for Castleman disease Hôpital Saint Louis Université de Paris Paris France; ^12^ INSERM U976 HIPI, Hôpital Saint Louis Université de Paris Paris France

**Keywords:** Castleman disease, chronic venous disease, lymphedema

## Abstract

Idiopathic multicentric Castleman disease (iMCD) is a lymphoproliferative disease of unknown etiology. Deciphering mechanisms involved in CD pathogenesis may help improving patients’ care. Six cases of stereotyped sub‐diaphragmatic iMCD affecting lower limb‐draining areas and associated with severe and often ulcerative lower extremity chronic dermatological condition were identified in our cohort. Pathological examination revealed mixed or plasma‐cell type MCD. In three patients, shotgun metagenomics failed to identify any pathogen in involved lymph nodes. Antibiotics had a suspensive effect while rituximab and tocilizumab failed to improve the condition. This novel entity requires a specific approach and exclusion of potentially harmful immunomodulation.

## 1 INTRODUCTION

Castleman disease (CD) is a rare lymphoproliferative disorder defined by peculiar pathological features ranging from the hyaline‐vascular type with regressed germinal centres and hypervascularization to the plasma cell type with intense interfollicular plasma cell infiltration [1]. Three forms of the disease include the unicentric form with a usually asymptomatic single lymph node and two multicentric forms (multicentric Castleman disease [MCD]) often associated with inflammatory symptoms, hypergammaglobulinemia and fluid overload [2]. Some of these MCD cases are related to a polyclonal proliferation of human herpesvirus 8 (HHV8)‐infected plasmablasts (HHV8+ MCD) [3], and the remaining cases are considered as 'idiopathic' (idiopathic MCD [iMCD]) [4, 5, 6]. Although an infectious origin of iMCD has been thoroughly sought, no causative infectious agent has been identified to date despite the use of high‐throughput sequencing technologies [7]. We here describe six cases of iMCD associated with severe venous or lymphatic disease of the lower limbs. This novel subtype of MCD must be recognized as it requires specific treatment including prolonged antibiotics and specific care of the underlying venous/lymphatic disease.

## 2 METHODS

### 2.1 Identification of the cases

Patients were identified in a cohort of 66 iMCD patients belonging to the French national reference centre for CD. All files were reviewed by a group of clinicians and pathologists with an expertise in the field of CD (DB, EO, RB, LG, VM, EP, AM). All patients fulfilled currently satisfying criteria for idiopathic MCD, including clinical, biological and histopathological criteria, as described in Fajgenbaum et al. [6] All patients gave informed consent for the research, and the project was reviewed and approved by local ethic committee.

### 2.2 Shotgun metagenomic analysis of three MCD samples

This aspect has been detailed in the Supplementary data section.

## 3 RESULTS

### 3.1 Detailed description of the first patient (P1)

**FIGURE 1:**
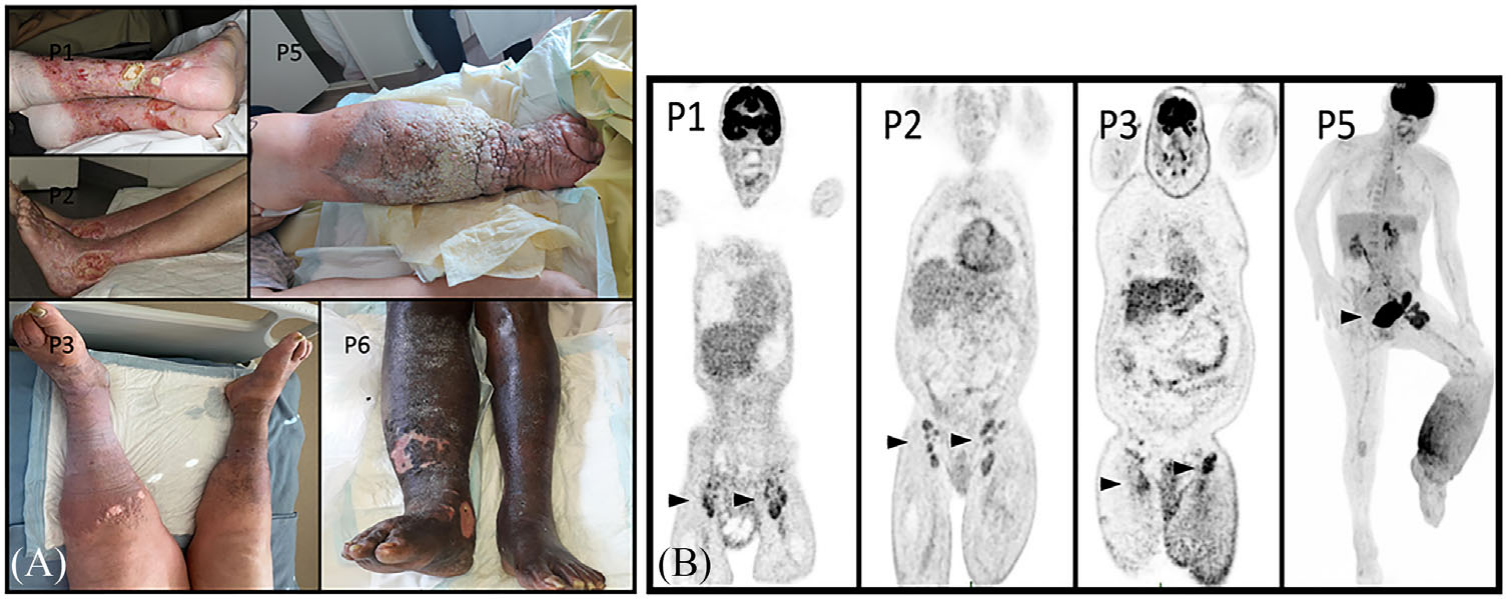
Clinical and metabolic aspects of leg‐type idiopathic multicentric castleman disease (iMCD). (A) Clinical aspects of lower limb lesions in patients with leg‐type MCD (P1, P2, P3 P5 and P6). (B) Metabolic aspects of skin and lymph node lesions in patients with leg‐type MCD (P1, P2, P3 and P5), black arrows underline ilio‐inguinal hypermetabolic involvement

A 67‐year‐old male, with a medical history of pulmonary tuberculosis and aspergilloma, was referred to our hospital for the management of chronic venous leg ulcers (Figure 1A), associated with recent bilateral inguinal and non‐compressive lymphadenopathy (short‐axis diameter = 30 mm). He presented with lipodermatosclerosis and ulcers since 2003, and no remission had been obtained despite prolonged local treatment and elastic compression. He recently reported inflammatory symptoms associated with the occurrence of painless enlarged lymph nodes in the inguinal area. No peripheral neuropathy was noted. Lymph node biopsy was consistent with a diagnosis of mixed‐type CD. HHV8 latency associated nuclear antigen 1 (LANA‐1) staining was negative. Biological evaluation showed high levels of CRP, elevated serum vascular endothelial growth factor (VEGF) and marked polyclonal hypergammaglobulinemia (Table 1). No monoclonal component was found. The patient was tested Human Immunodeficiency Virus (HIV) negative. There were no clinical or biological features suggestive of autoimmune disease. Positron emission tomography computerized tomography (PET‐CT) (Figure 1B) showed moderately hypermetabolic (SUVmax = 2.8) bilateral inguinal lymphadenopathy. Histopathological analysis of skin lesion biopsy found a large ulceration with vascular hyperplasia and polymorphic inflammatory infiltrate within the dermis compatible with a chronic ulcer disease without any evidence for vasculitis or plasma cell infiltration. He presented with clinical signs of infected skin ulcers. Skin cultures were positive for *Streptococcus agalactiae* and *Pseudomonas aeruginosa*, and adapted antibiotics were started. All symptoms quickly improved with amelioration of general status, local inflammatory signs, disappearance of lymphadenopathy, normalisation of CRP and decrease of serum gammaglobulin level. Shotgun metagenomics failed to identify any pathogen in the involved lymph node. After antibiotics cessation, he presented with several relapses of ulcer infection and responded to iterative administration of antibiotics. He finally died from septic shock related to a novel episode of *P. aeruginosa* skin superinfection.

**TABLE 1:** Characteristics of patients with leg‐type iMCD associated with venous/lymphatic disease

	Patient 1	Patient 2	Patient 3	Patient 4	Patient 5	Patient 6
Sex/Age (years)	M/67	M/65	M/60	M/64	F/38	M/74
Medical History	Pulmonary tuberculosis, aspergilloma	Myocardial infarction, chronic venous insufficiency	Morbid obesity, chronic venous insufficiency	Morbid obesity, type 2 diabetes, chronic venous insufficiency, chronic cardiac failure	Congenital lymphedema, pulmonary tuberculosis	Type 2 diabetes, obesity, alcoholism
Delay for MCD diagnosis (years)	16	5	4	NA	13	NA
Hb (g/dl)	8.6	9.5	13.5	9.6	10.5	9.5
Platelets (G/L)	476	230	216	216	611	227
Lymphocytes (G/L)	1.89	1	1.94	1.49	1.43	2.1
CRP (mg/L)	79	62	83	13	112	84
Gammaglobulins (g/L)	33	21.9	26.2	17.7	24	33
Serum IL‐6 (pg/ml)	NA	NA	NA	NA	37.6	NA
VEGF (pg/ml)	1064	NA	415	NA	1109	726
Auto‐antibody panel	Negative	Negative	Negative	Negative	Negative	Negative
PET‐CT	II	II	II	NA	I	NA
Stage/SUVmax	2.8	3.5	5		6.34	
Lymph node pathology Microbial shotgun metagenomics	Mixed‐type CD	Mixed‐type CD	Mixed‐type CD	Mixed‐type CD	Plasma‐cell type CD	Mixed‐type CD
	Negative	Negative	Negative	NA	NA	NA
					
Skin lesion Skin pathology Bacteriological cultures	Venous ulcers of the lower limbs with recurrent erysipelas Chronic venous ulcer *Streptococcus agalactiae* *Pseudomonas aeruginosa*	Mixed arteriovenous ulcers of the lower limbs with recurrent erysipelas Superficial fibrosis with non‐specific cellular infiltrate *Pseudomonas aeruginosa*, *Pasteurella multocida*	Bilateral lymphedema with recurrent erysipelas NA NA	Bilateral lymphedema, mixed arteriovenous ulcers of the lower limbs No episode of superinfection NA *Pseudomonas aeruginosa* *Staphylococcus aureus*	Left leg lymphedema with recurrent erysipelas Non‐specific fibro‐inflammatory lesions *Pseudomonas aeruginosa*, *Staphylococcus aureus*, *Streptococcus dysgalactiae*	Right leg lymphedema, mixed arteriovenous ulcers with one episode of erysipela NA NA
Treatment	Repeated antibiotics	Tocilizumab Repeated antibiotics	Rituximab Repeated antibiotics	None	Repeated antibiotics, transfemoral amputation	Long‐term antibiotics
Outcome	Death/Cellulitis	Death/Unknown cause	Stable disease	NA	Stable disease	Death/Respiratory failure

Abbreviations: CRP, C‐reactive protein; CT, computerized tomography; Hb, haemoglobin; IL‐6, interleukin 6; MCD, multicentric Castleman disease; NA, not available; PET, positron emission tomography; SUV, standardized uptake value; VEGF, vascular endothelial growth factor.

* Auto‐antibody panel included screening for antinuclear antibody, anti‐double stranded DNA (dsDNA), anti‐extractable nuclear antigen (ENA), anti‐cyclic citrullinated peptide (CCP), rheumatoid factor (RF) and anti‐neutrophil cytoplasmic antibodies (ANCA).

### 3.2 Patients’ clinical and biological characteristics (P1 to P6)

Between August 2000 and August 2021, six patients diagnosed with iMCD presented with similar characteristics. Five of the six patients were male, and age at iMCD diagnosis ranged from 38 to 74 years. The onset of the skin condition preceded the diagnosis of MCD in all cases, with an average duration of 9.5 years. Three patients reported inflammatory symptoms including fatigue, fever or weight loss. Cutaneous pre‐existing conditions were chronic venous ulcers in four, congenital lymphedema in one and obesity‐related lymphedema in three (Figure 1A). Short‐axis diameter of the involved lymph nodes ranged from 30 to 90 millimeters. Biological markers showed an inflammatory syndrome with elevated serum CRP levels (range, 13–112 mg/L) and polyclonal hypergammaglobulinemia (range, 17.7–33 g/L) in all patients, anaemia in four and elevated serum VEGF levels in the four patients tested. No monoclonal component was isolated. Screening for autoimmunity, HHV8 or HIV infection was negative. (Table 1).

PET‐CT was available in four patients and identified bilateral (*n* = 3) or unilateral (*n* = 1) hypermetabolism involving inguinal and iliac lower‐limb draining lymph nodes (SUVmax ranging from 2.8 to 6.3) (Figure 1B).

Histopathological examination of the affected lymph nodes was consistent with mixed‐type CD in five cases and plasma‐cell type in one. Mixed‐type iMCD encompassed both aspects of hyaline‐vascular (regressed germinal centres, follicular dendritic cell expansion and increased vascularity) and plasma‐cell (intense interfollicular plasmacytosis and hyperplastic germinal centres) iMCD variants, as reported in Fajgenbaum et al. [6] and described in Table S1 and Figure S1. LANA staining was negative in all patients. Lymph node shotgun metagenomic analysis was performed on three samples and failed to identify any infectious agent.

### 3.3 Review of the literature

Review of the literature identified two additional patients with a similar presentation associating lymphedema, infected venous ulcers with repeated erysipelas and regional MCD. A surgical approach with extended removal of the lymphedema led to clinical improvement in the first patient but biological parameters and long‐term follow‐up were not available [8]. Remission was obtained in the second patient after skin resection and graft [9].

### 3.4 Disease course and treatment

Five patients were treated with antibiotics and local care, which led to transitory and partial clinical and biological improvement, but numerous episodes of relapsing skin superinfection occurred. Two patients (P2, P3) received a conventional iMCD treatment: Patient 2 received one dose of intravenous tocilizumab complicated by *Pasteurella multocida* septicemia, and the treatment was stopped; patient 3 failed to respond to a 4‐weekly course of rituximab. Patient 4 was lost to follow‐up. Patient 5 required a left transfemoral amputation. Lymphadenopathy and hypergammaglobulinemia persisted despite the resolution of the inflammatory syndrome and no sign of superinfection. The persistent enlarged lymph nodes prevent the fitting of a suitable prothesis. Patient 6 died of respiratory failure.

## 4 DISCUSSION

We here described six cases of stereotyped sub‐diaphragmatic iMCD affecting lower limb‐draining areas and associated with severe and often ulcerative lower extremity chronic dermatological condition (chronic lymphedema and/or chronic venous insufficiency). Two similar observations were identified by reviewing the literature. Taken together, these observations appear to identify a subtype of iMCD, we suggest denominating 'leg‐type' CD and requiring specific attention. This severe condition accounts for 9% of iMCD cases in our cohort. The link between leg lesions and the occurrence of CD is unknown. One can hypothesize that chronic venous and/or lymphatic insufficiency, promoting the occurrence of infectious flares, may lead to CD lesions through chronic antigen stimulation in the draining lymph nodes. The fact that some patients improved under antibiotic treatment is in line with this downstream hypothesis, even if in situ shotgun metagenomic analysis of the lymph nodes was negative in the three patients tested. The absence of microorganisms identified from the lymph nodes suggests the purely reactive nature of leg‐type iMCD. Antimicrobial treatment or radical surgery only partially improved the picture, an observation consistent with the persistence of an autonomous immunopathogenic process. Interestingly, one patient did not show any sign of infection despite bacterial colonization of the skin lesions. On the other hand, one could hypothesize that the CD lesion is at the origin of the syndrome. Skin lesions preceded the onset of nodal disease by years, and we failed to demonstrate any compression of the local vascular structures by the enlarged lymph nodes that would favour an upstream hypothesis of this syndrome. It is noteworthy that some cases of iMCD present with fluid overload, edema and high levels of serum VEGF as in three patients from the present series [10, 11]. We believe that this novel entity needs to be clearly identified by clinicians because conventional treatment of iMCD based on immunomodulatory drugs [11, 12] failed to improve patients’ condition and might even be harmful, as demonstrated by *Pasteurella* bacteriemia following tocilizumab therapy in patient 2. We therefore propose specific treatment guidelines for patients with leg‐type MCD, such as prolonged treatment of infectious flares or long‐term antibioprophylaxis, in association with treatment of the underlying venous or lymphatic condition when possible.

## CONFLICT OF INTEREST

E. Oksenhendler is a consultant for Eusapharma. The other authors have no conflict of interest to disclose.

## AUTHOR CONTRIBUTIONS

T. Ballul and D. Boutboul wrote the manuscript. T. Ballul, D. Boutboul, L. Galicier, L. Maisonobe, J. Fadlallah, N. Belfeki, R. Bertinchamp, M. Jachiet, M. Garzaro, M. Malphettes, C. Fieschi, H. Guillot, H. Bensekhri, A. de Masson and E. Oksenhendler contributed to the patient recruitment and management. J. Poirot prepared the lymph nodes samples. V. Meignin, A. de Masson and E. Poullot reviewed lymph node pathology. P.‐L. Woerther and A. Martin performed the shotgun metagenomic assays and analysed the data. D. Boutboul supervised the project. All the authors reviewed the manuscript.

## Supporting information

Supporting Information

Supporting Information

Supporting Information
