# Supplementary material for: Leg‐type form of idiopathic multicentric Castleman disease associated with severe lower extremity chronic venous/lymphatic disease
Source: EJHaem. 2021 Dec 23;3(1):175–9. doi: 10.1002/jha2.353 (PMC9175857; doi:10.1002/jha2.353)
Supplement: Supplementary file 1 — Supporting Information [file JHA2-3-175-s002.docx]

**SUPPLEMENTARY MATERIAL (Ballul et al.)**

*Shotgun metagenomic analysis of three MCD samples*

Three lymph node samples from patients 1, 3 and 5 kept at -80°C were used for unbiased shotgun metagenomics analysis. Pre-extraction by bead beating combined with chemical cell disruption were followed by extraction using QiaSymphony (Qiagen, Hilden, Germany). Both a negative control and a positive control containing bacteria (including Gram-positive and Gram-negative aerobic and anaerobic species), viruses (including enveloped and non-enveloped RNA and DNA viruses), and fungi (including filamentous and non-filamentous pathogens) were tested in each run. DNA and RNA libraries were prepared in parallel using Nextera XT DNA (Illumina, San Diego, CA, USA) and the Human RiboZero TruSeq Stranded Total RNA Library Prep Kit (Illumina, San Diego, CA, USA), respectively. The quality of each library was evaluated using a D1000 ScreenTape on a TapeStation (Agilent, Santa Clara, CA, USA) and the quantity using the Quant-it dsDNA Assay kit (Thermo Fischer, Waltham, MA, USA) on a Varioskan Lux (Thermo Fischer, Waltham, MA, USA). The DNA and RNA libraries were tagged and normalized to equal concentrations before pooling, denaturation, and pair-end sequencing using the High Output Kit v2, 2x150 bp on a NextSeq500 Illumina device (Illumina, San Diego, CA, USA). RNA and DNA sequences were analysed separately using the in-house MetaMIC® software (IDDN.FR.001.160012.000.S.C.2018.000.31230). The pair-end sequences were retained for the final count of identified microorganisms only if R1 and R2 files were concordant with the same reference. Human sequences were removed using the hg19 database (Full data set GRCh37/hg19, feb 2009), as well as sequences with insufficient qualityes (Phred scores < 20). The identification of non-human sequences were performed using a cleaned NCBI nt and nr (Genbank release 237, Apr 2020) database, which contains all known microbes, as well as an in-house bacterial, fungal, and viral database. For each sample, the negative control sequences were subtracted from those of the samples after normalization of the number of corresponding sequences to the total number of sequences.
