# Supplementary figures and images for: Leg‐type form of idiopathic multicentric Castleman disease associated with severe lower extremity chronic venous/lymphatic disease
Source: EJHaem. 2021 Dec 23;3(1):175–9. doi: 10.1002/jha2.353 (PMC9175857; doi:10.1002/jha2.353)

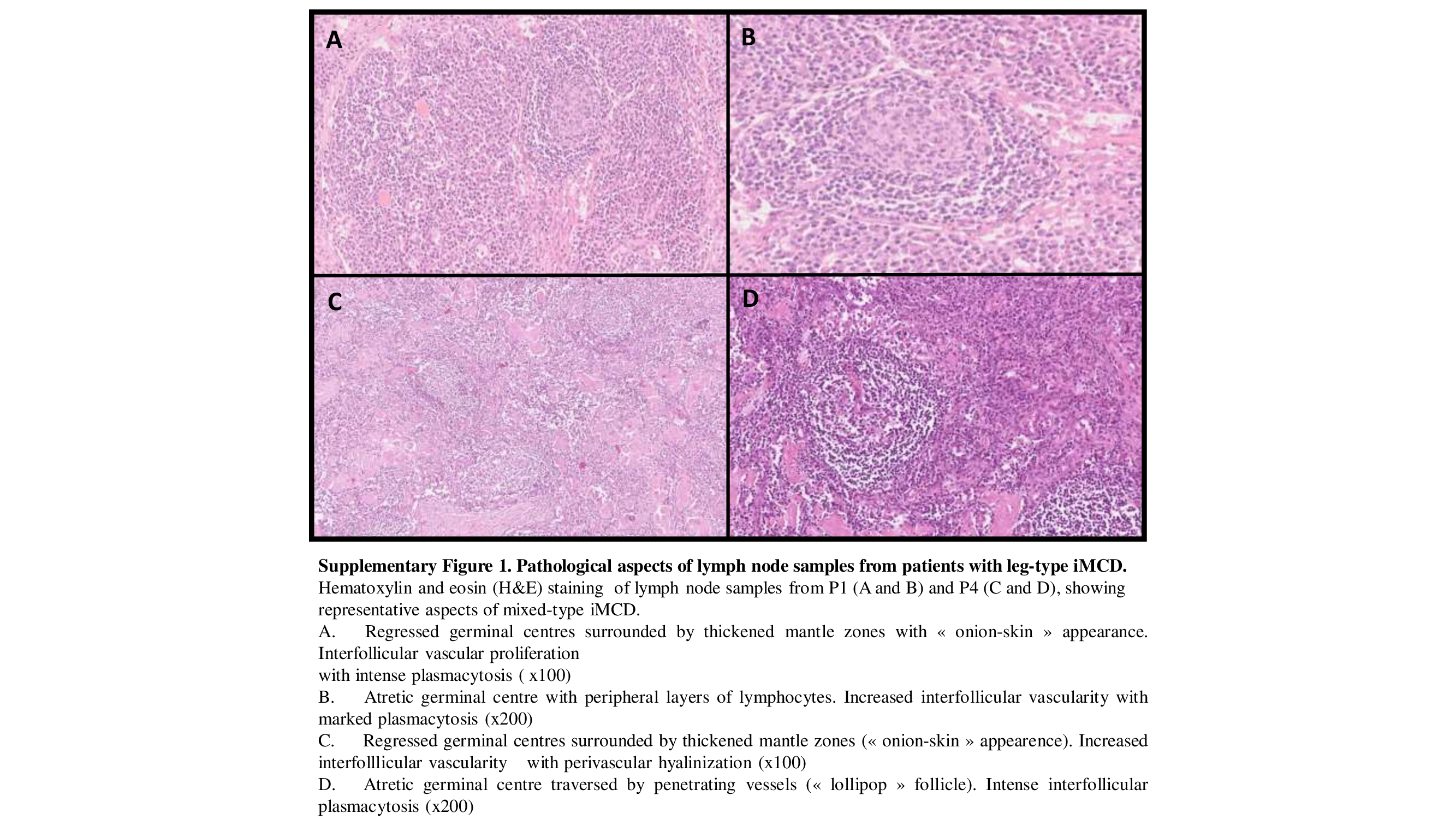

Supplement: Supplementary file 2 — Supporting Information [file JHA2-3-175-s003.tiff]
