# Supplementary material for: Leg‐type form of idiopathic multicentric Castleman disease associated with severe lower extremity chronic venous/lymphatic disease
Source: EJHaem. 2021 Dec 23;3(1):175–9. doi: 10.1002/jha2.353 (PMC9175857; doi:10.1002/jha2.353)
Supplement: Supplementary file 3 — Supporting Information [file JHA2-3-175-s001.docx]

| Re | **Regressed germinal centres** | **Follicular dendritic cell prominence** | **Vascularity** | **Hyperplastic germinal centres** | **Plasmacytosis** |
| --- | --- | --- | --- | --- | --- |
| **P1** | +++ | +++ | +++ | - | ++ |
| **P2** | ++ | ++ | ++ | - | +++ |
| **P3** | + | + | ++ | ++ | +++ |
| **P4** | ++ | ++ | +++ | ++ | + |
| **P5** | - | + | ++ | ++ | +++ |
| **P6** | ++ | + | ++ | + | +++ |

**Supplementary Table 1. Grading of pathologic features observed in patients with leg-type iMCD**

*A semi-quantitative scale ranging from – (absent) to +++ (very prominent) has been used to describe the different features observed in patients’ lymph nodes.*
